# Odontogenic infection by *Porphyromonas gingivalis* exacerbates fibrosis in NASH via hepatic stellate cell activation

**DOI:** 10.1038/s41598-020-60904-8

**Published:** 2020-03-05

**Authors:** Atsuhiro Nagasaki, Shinnichi Sakamoto, Chanbora Chea, Eri Ishida, Hisako Furusho, Makiko Fujii, Takashi Takata, Mutsumi Miyauchi

**Affiliations:** 1 Department of Oral and Maxillofacial Pathobiology, Graduate School of Biomedical and Health Sciences, Hiroshima University, Hiroshima, Japan; 2 Department of Advanced Prosthodontics, Graduate School of Biomedical and Health Sciences, Hiroshima University, Hiroshima, Japan; 3 Department of Global Dental Medicine & Molecular Oncology, Graduate School of Biomedical and Health Sciences, Hiroshima University, Hiroshima, Japan; 4 Tokuyama University, Tokuyama, Japan

**Keywords:** Transforming growth factor beta, Infection, Bacterial infection

## Abstract

Odontogenic infection of *Porphyromonas gingivalis* (*P.g*.), a major periodontal pathogen, exacerbates pathological progression of non-alcoholic steatohepatitis (NASH). In this study, we aimed to clarify the detailed mechanism in which *P.g*. induced hepatic stellate cells (HSCs; key effector cells in liver fibrosis) activation. In the liver of high fat diet-induced NASH mouse model with *P.g*. odontogenic infection, immunolocalization of *P.g*. was detected. The number of hepatic crown-like structure, which was macrophage aggregation and related to liver fibrosis, was drastically increased and fibrosis area was also increased through upregulating immunoexpression of Phosphorylated Smad2 (key signaling molecule of TGF-β1) and Galectin-3. *P.g*.-secreted trypsin-like enzyme [gingipain; an activator of protease-activated receptor 2 (PAR2)] stimulated HSC proliferation and differentiation through Smad and ERK signaling induced by TGF-β1 produced from HSCs with *P.g*.-infection. Further, Galectin-3 produced from HSCs with *P.g*. infection and *P.g*.-derived LPS/lipoprotein stimulation stabilized TGFβ-receptor II resulting in increasing sensitivity for TGF-β1, finally leading to HSC differentiation via activating Smad and ERK signaling. In addition to them, hepatocytes (main component cells of liver) contributed to HSC activation through TGF-β1 and Galectin-3 production in paracrine manner. Collectively, *P.g*.-odontogenic infection exacerbates fibrosis of NASH by HSC activation through TGF-β1 and Gal-3 production from HSCs and hepatocytes.

## Introduction

Non-alcoholic fatty liver disease (NAFLD) is a chronic hepatic disease caused by obesity including simple steatosis and non-alcoholic steatohepatitis (NASH). The morbidity rate of NAFLD is high, up to 30% in Western countries, with increasing the number of NAFLD patients because of the increase of obesity^1–3^. Most NAFLD patients indicate simple steatosis, which is a reversible condition, but 10–20% of simple steatosis progresses to NASH, which is presented as inflammation with hepatocyte degeneration followed by hepatic fibrosis^3^. As some of the NASH cases eventually result in liver cirrhosis and cancer, it is a critical health problem requiring adequate prevention and early therapeutic intervention^2,4^. The mechanisms for the development and progression of NASH are extremely complicated. Recently, it was reported that fat deposition, inflammation and fibrosis in NASH are simultaneously caused by many factors such as up-regulation of free fatty acids (FFA), oxidation stress, cytokines and bacterial lipopolysaccharide (LPS)^5^.

*Porphyromonas gingivalis* (*P.g*.) is a main periodontal pathogen. Periodontitis caused by *P.g*. is a well-recognized risk factor for many systemic diseases such as cardiovascular disease, preterm birth, diabetes mellitus, and rheumatoid arthritis^6–9^. *P.g*. can enter the blood circulation from periodontal disease sites and disseminate into the whole body and it can be detected in the distant organs including liver^6,7,10,11^. Furusho *et al*. reported that *P.g.-*odontogenic infection exacerbated pathological progression, especially the fibrosis stage of NASH, using a high fat diet (HFD)-induced NASH mouse model, which *P.g*. was detected in the liver with increased serum LPS^10^. It is known that main virulence factors of *P.g*. are pathogen-associated molecular pattern molecules (PAMPs) and gingipain (a trypsin like enzyme). PAMPs bind to specific receptors such as toll-like receptors (TLRs) and resulting in inducing periodontal inflammation^12^. While gingipain activate protease-activated receptor2 (PAR2) leading to transforming growth factor-β1 (TGF-β1) production from gingival fibroblast^13^. Interestingly, our group has demonstrated that HFD-induced NASH mouse model significantly increased toll-like receptor 2 (TLR2) expression in liver, suggesting increasing reactivity for *P.g.-*derived PAMPs^10^. However, the detailed mechanisms of liver fibrosis caused by *P.g.-*odontogenic infection are still unclear.

Recently, hepatic stellate cells (HSCs) have attracted interest as effector cells of fibrosis after inflammation^14,15^. Under pathological conditions, HSCs proliferate and differentiate to myofibroblastic cells with extracellular matrix production (HSC activation), resulting in liver fibrosis. Furthermore, TGF-β1 and Galectin-3 (Gal-3; a unique chimera-type β-galactoside-binding protein of the galectin family) are reported to be the key molecules for liver fibrosis through HSC activation^14,16,17^. Several signaling pathways and molecules for hepatic fibrosis have been identified including TGF-β1/Smad and /ERK signaling pathways as the major pathways for the activation of HSC that lead to up-regulation of the markers of liver fibrosis such as a-smooth muscle actin (α-SMA) and type I collagen^14,16–19^. Gal-3 is also required for HSC activation^16,20^. It was reported that engulfment of apoptotic bodies (dead hepatocyte) increased Gal-3 production from HSC and activated HSC in autocrine manner. Interestingly, Gal-3 knock-out mice displayed significantly decreased liver fibrosis with reduced expression of *Tgfβ1*, *Acta2* and *Col1a1* vs. WT following bile duct-ligated to induce experimental live fibrosis, suggesting that Gal-3 also played important roles in liver fibrosis^16^. Further, it was suggested that Gal-3 cross-linked N-glycans on TGF-β receptors at cell surface and delayed its removal by constitutive endocytosis^21^. Thus, Gal-3 is a key molecule for HSC activation and liver fibrosis by cooperating with TGF-β1.

In this study, to shed light on the mechanism underlying the progression of liver fibrosis caused by *P.g.-*odontogenic infection, the roles of HSC activation caused by *P.g.-* infection through gingipain and *P.g*.-derived LPS/lipoprotein stimulations in the process of liver fibrosis have been analyzed, especially focusing on TGF-β1 and Gal-3.

## Results

### Periapical granuloma is the persist source of *P.g*

**Figure 1:**
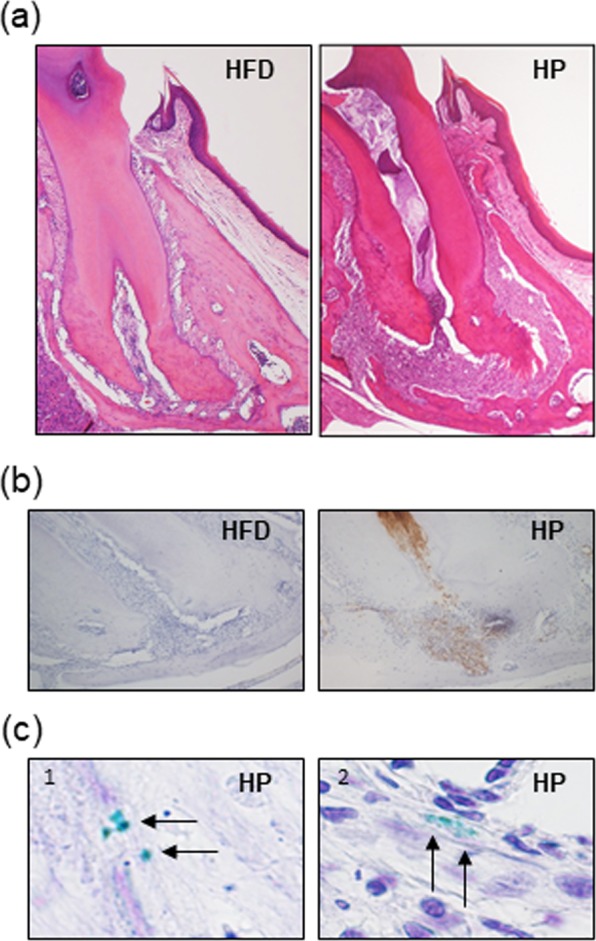
*P.g*.-odontogenic infection induces inflammation in jaw. (**a**) Histological findings of root apex area at 9 weeks after *P.g*.-odontogenic infection. After 9 weeks of *P.g*.-odontogenic infection, periapical granuloma was seen at the root apex area of infected tooth (H&E staining). Magnification: X40. (**b**) Immunohistochemistry of neutrophils in the dental granuloma. Numerous Ly-6B.2 positive neutrophils (brown granules) are observed in HP. Magnification: X100. (**c**) Immunohistochemically *P.g*. was detected in necrotic pulp and viable cells in periapical granuloma. Magnification: X1000. Arrow: *P.g*.

There was no significant difference in body weights among groups during the experimental period [Supplementary Fig. 1]. At 9 weeks of *P.g.-*infection, pulp necrosis and dental granuloma at the root apex area were observed (HP) [Fig. 1a]. Accumulation of neutrophils in dental pulp and granuloma around the tooth apex were detected [Fig. 1b]. Immunolocalization of *P.g*. was observed in necrotic pulp [Fig. 1c1] and periapical granuloma [Fig. 1c2].

### *P.g.-*odontogenic infection exacerbates pathological progression of NASH through Gal-3 and TGF-β1/Smad pathway

**Figure 2:**
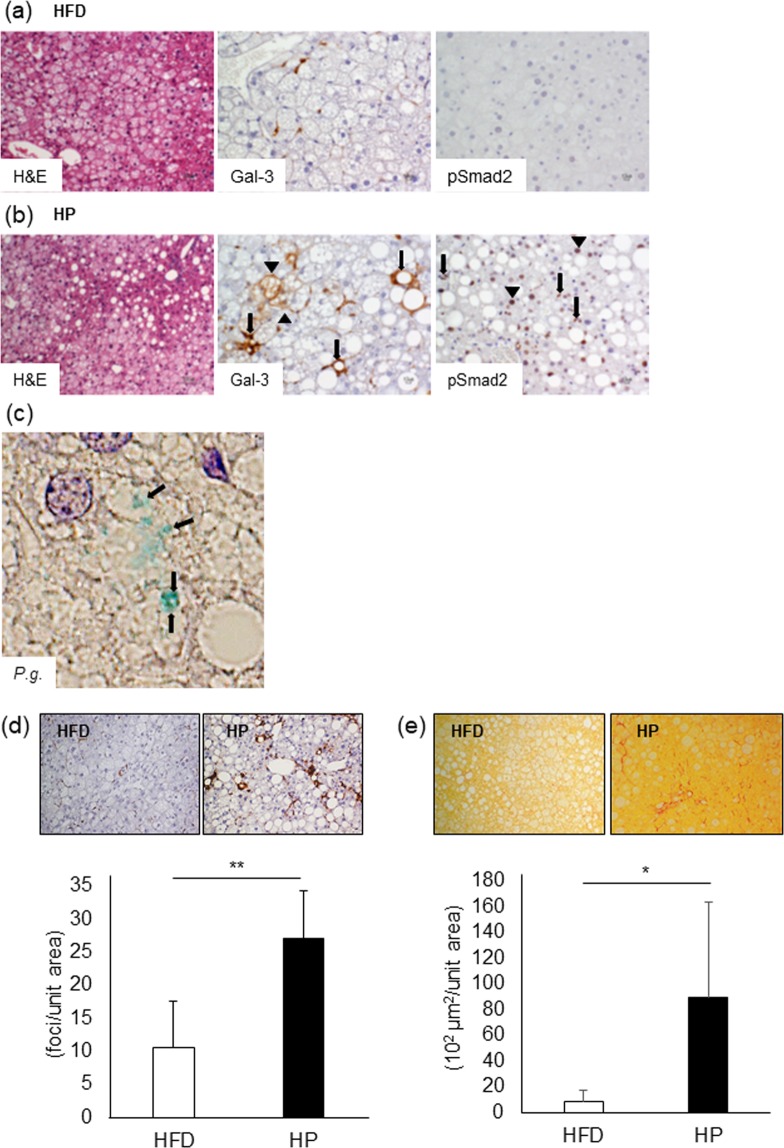
*P.g*.-odontogenic infection promotes liver fibrosis in model mice. The liver in HFD group (**a**) and HP group (**b**) were histologically analyzed including H&E staining (Magnification x100) and immunohistochemistry. Expression of Gal-3 and pSmad2 were analyzed immunohistochemically. Gal-3; Arrow: hCLS. Arrowhead: Gal-3 expressing hepatocyte. pSmad2; Arrow: HSC. Arrowhead: hepatocyte. Magnification: X200. (**c**) *P.g*. was detected in hepatocytes with *P.g*.-odontogenic infection. Arrow: *P.g*. Magnification: X1000. (**d**) hCLS was immunohistochemically detected as accumulation of Gal-3-positive macrophages. Magnification: X200. The number of hCLS was counted and number/unit area was calculated. (**e**) The red-stained fibrosis area was measured, and area/unit area was calculated. Magnification: X200. HFD = High Fat Diet (N = 5), HP = HFD + *P.g.-*infection (N = 5). Results were shown as mean ± SD. *p < 0.05, **p < 0.01. Gal-3: Galectin-3.

Figure 2 shows histological changes in HFD [Fig. 2a] and HP liver tissues [Fig. 2b]. In HFD group, microvesicular lipid deposition was prominent, but inflammation was slight. Whereas increasing macrovesicular lipid accumulation and hepatic crown-like structures (hCLS), which was aggregation of macrophages and positively correlated with the extent of liver fibrosis [Supplementary Fig. 2], were seen in HP group. Immunoexpression of Gal-3 and pSmad2 (a key signaling molecule of TGF-β1), which were critical molecules for HSC activation, was examined. Among hepatocytes in HFD, Gal-3 positive spindle cells were scattered. While in addition to increasing Gal-3 positive spindle cells, Gal-3 positive hCLS (arrows) and hepatocytes (arrowheads) were observed. Strong pSmad2 nuclear expression, indicating TGF-β1 signaling activation, was detected in HSCs (arrows) and hepatocytes (arrowheads) of HP group. HFD group showed negative or weak reaction for pSmad2. Interestingly, *P.g*. was clearly detected in liver of HP group [Fig. 2c]. Morphometrically, the number of Gal-3 positive hCLS was counted. The number of hCLS in HP group was significantly increased (*p* < 0.01), [Fig. 2d]. To analyze the degree of liver fibrosis, sirius red staining was performed. Sirius red-positively stained collagen fibers are distributed among hepatocytes with lipid deposition. HP group indicated significantly increased sirius red positive fibrosis areas (*p* < 0.05), [Fig. 2e]. These data suggest that *P.g.-*odontogenic infection aggravates inflammatory cell infiltration and liver fibrosis, in which TGF-β1/Smad and Gal-3 pathway are involved.

### *P.g.-*infection and LPS-PG (*P.g*.-LPS/lipoprotein) stimulation induce HSC activation

**Figure 3:**
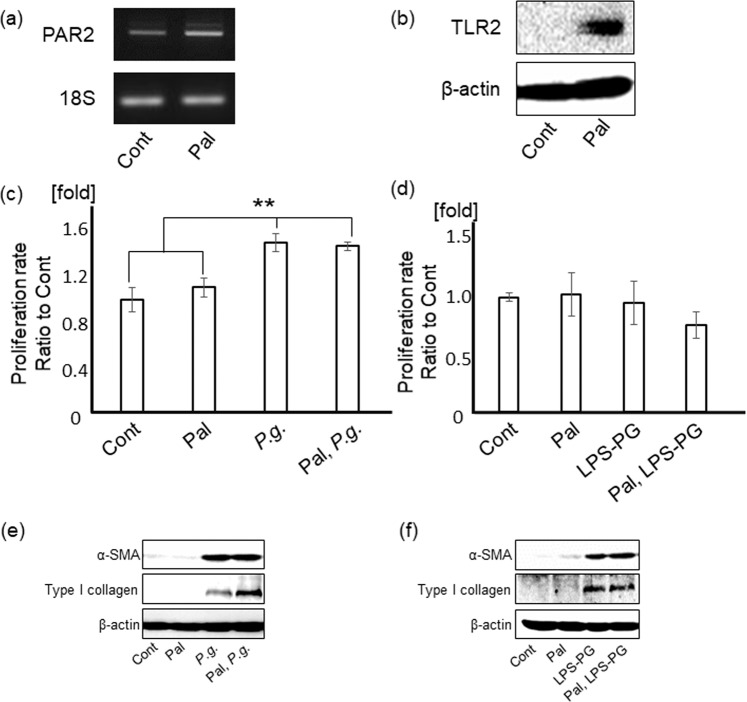
*P.g*.-infection and LPS-PG induce myofibroblastic activation of HSCs. PAR2 (**a**) and TLR2 (**b**) expressions in LX-2 cells with/without palmitate treatment were analyzed with RT-PCR and western blotting, respectively. (**c**) LX-2 cells with/without palmitate treatment were cultured with *P.g*. (MOI-100) for 5 days and counted by a coulter counter. Cont/Pal/*P.g*./Pal, *P.g*.: N = 5. (**d**) LX-2 cells with/without palmitate treatment were cultured with 1 µg/ml of LPS-PG for 5 days and counted by coulter counter. Cont/Pal, LPS-PG: N = 4, Pal/LPS-PG: N = 3. (**e**) LX-2 cells with/without palmitate treatment were cultured with *P.g*. (MOI-100) for 5 days. α-SMA and type I collagen were detected by western blotting. (**f**) LX-2 cells with/without palmitate treatment were cultured with LPS-PG (1 μg/ml) for 6 days. α-SMA and type I collagen were detected by western blotting. 18S was used as internal control for RT-PCR and β-actin was used as internal control for western blotting. Results were shown as mean ± SD. **p < 0.01. Pal: palmitate, *P.g*.: *P.g.-*infection.

It is well accepted that PAR2 is activated by trypsin-like enzymes and contributes to TGF-β1 production which is resulting in liver fibrosis^22^. Gingipain; a major virulence factor of *P.g*., is known to activate PAR2^13,23,24^. To determine the effects of *P.g*.-LPS/lipoprotein, LPS-PG, a ligand for both TLR2 and 4, was used in this study. Palmitate (a main FFA upregulated in serum of NASH patient) treatment significantly upregulated PAR2 [Fig. 3a] and TLR2 levels [Fig. 3b], but not toll-like receptor 4 (TLR4) level [Supplementary Fig. 3a] in LX-2 cells, human hepatic stellate cell line.

*P.g-*infection significantly promoted proliferation of LX-2 cells with/without palmitate treatment (*p* < 0.01), [Fig. 3c]. Whereas LPS-PG stimulation showed no change in proliferation of LX-2 cells [Fig. 3d].

In LX-2 cells with/without palmitate, α-SMA and type I collagen as markers of myofibroblastic differentiation of HSCs, were prominently upregulated by both *P.g.-*infection [Fig. 3e] and LPS-PG stimulation [Fig. 3f].

### Stimulation of PAR2-TGF-β1 pathway caused by *P.g.-*infection induces HSC activation

**Figure 4:**
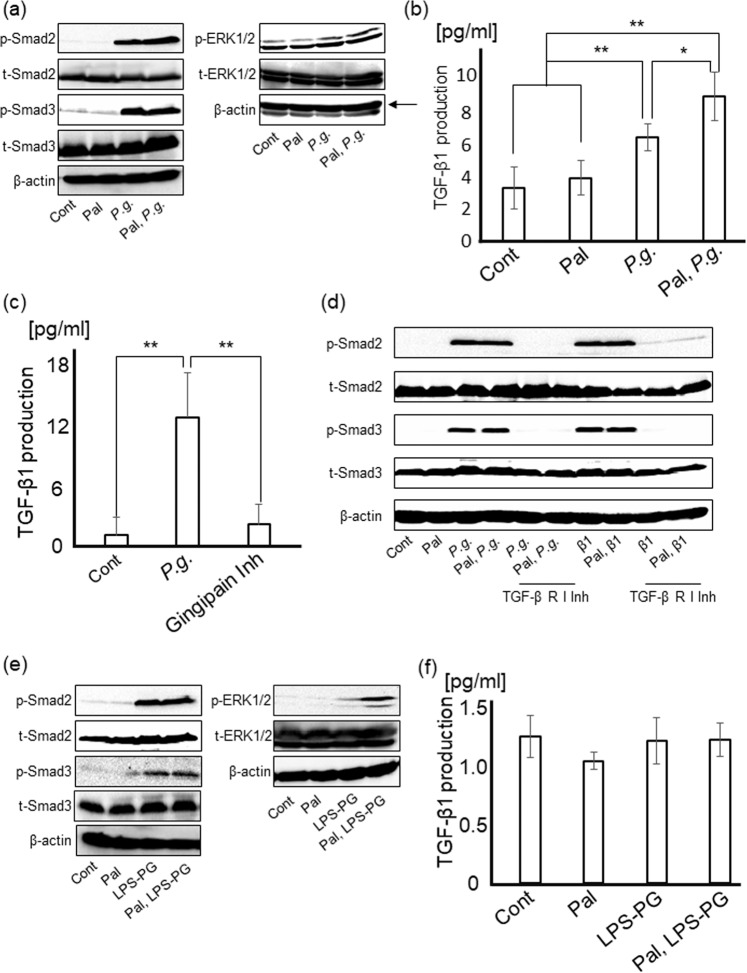
*P.g*.-infection and LPS-PG induce myofibroblastic activation of HSCs through Smad and ERK signaling pathways. *P.g*. infection induces TGF-β1 production through PAR2 activation by gingipain. (**a**) Smad2, Smad3, and ERK1/2 were detected by western blotting at 24 hours after *P.g.-*infection. (**b**) LX-2 cells with/without palmitate treatment were cultured with *P.g* (MOI-100) for 24 hours. The amount of TGF-β1 in each supernatant was measured by ELISA. Cont/Pal: N = 5, *P.g*./Pal, *P.g*.: N = 6. (**c**) LX-2 cells with/without gingipain inhibitors (3 µM) were cultured with *P.g*. (MOI-100) for 24 hours. Cont/Gingipain Inh: N = 8, *P.g*.: N = 7. (**d**) After culturing for 24 hours with/without TGF-β receptor I inhibitor (1 μg/ml), LX-2 cells were cultured with *P.g*. (MOI-100) for 24 hours. Smad2 and Smad3 were detected by western blotting. (**e**) Smad2, Smad3, and ERK1/2 were detected by western blotting at 4 days after LPS-PG stimulation. (**f**) LX-2 cells were cultured with LPS-PG (1 μg/ml) for 24 hours. The amount of TGF-β1 in each supernatant was measured by ELISA. Cont/Pal/LPS-PG/Pal, LPS-PG: N = 5. β-actin was used as internal control. Results were shown as mean ± SD. *p < 0.05, **p < 0.01. Pal: palmitate, *P.g*.: *P.g.-*infection, Gingipain Inh: Gingipain inhibitor, β1: TGF-β1, TGF-β R I Inh: TGF-β receptor I inhibitor.

In addition to Smad signaling, phosphorylation of ERK1/2, which is reported directly/indirectly to be upregulated by TGF-signaling, was also examined^14,16–19^. *P.g.-*infection induced activation of Smad2, Smad3, and ERK1/2. [Fig. 4a], indicating that *P.g.-*infection induces HSC activation through Smad and ERK pathways similar to direct stimulation with TGF-β1 [Supplementary Fig. 4a,b]. To clarify the contribution of TGF-β1 to *P.g.-*infection induced myofibroblastic differentiation of HSCs, the effects of *P.g.-*infection on the TGF-β1 levels in culture media were determined. LX-2 cells significantly produced TGF-β1 after *P.g*. infection (*p* < 0.01), especially TGF-β1 from palmitate-treated LX-2 cells was significantly increased than from non-treated cells (approximately 39.7%), (*p* < 0.05), [Fig. 4b].

It is known that TGF-β1 induced via PAR2 signaling promotes to fibrosis in the liver^25^. Interestingly, gingipain, a trypsin-like enzyme secreted by living *P.g*., activates PAR2 in gingival fibroblasts and induced cytokine production such as TGF-β1, IL-6, 8 and matrix metallopeptidase 2 (MMP-2) resulting in inflammation^13,23,24^. To elucidate the importance of gingipain-PAR2 axis on TGF-β1 production caused by *P.g.-*infection, gingipain inhibitors were used. Gingipain inhibitors significantly inhibited TGF-β1 production from LX-2 cells (*p* < 0.01), [Fig. 4c].

Moreover, TGF-β receptor I, which directly phosphorylates TGF-β1 signaling molecules, inhibitor completely suppressed phosphorylation of Smad2 and Smad3 caused by *P.g.-*infection [Fig. 4d].

In LX-2 cells with LPS-PG stimulation, Smad2, Smad3, and ERK1/2 were prominently activated as well as *P.g*.-infection [Fig. 4e]. Especially, ERK pathway was highly activated in palmitate-treated LX-2 cells, in which expression level of TLR2 was markedly upregulated. However, LPS-PG stimulation could not induce any change in TGF-β1 production from LX-2 cells, unexpectedly [Fig. 4f].

### Gal-3 production caused by *P.g.-*infection and -LPS stimulation enhanced myofibroblastic differentiation of HSC through upregulation of TGF-β receptor II expression

**Figure 5:**
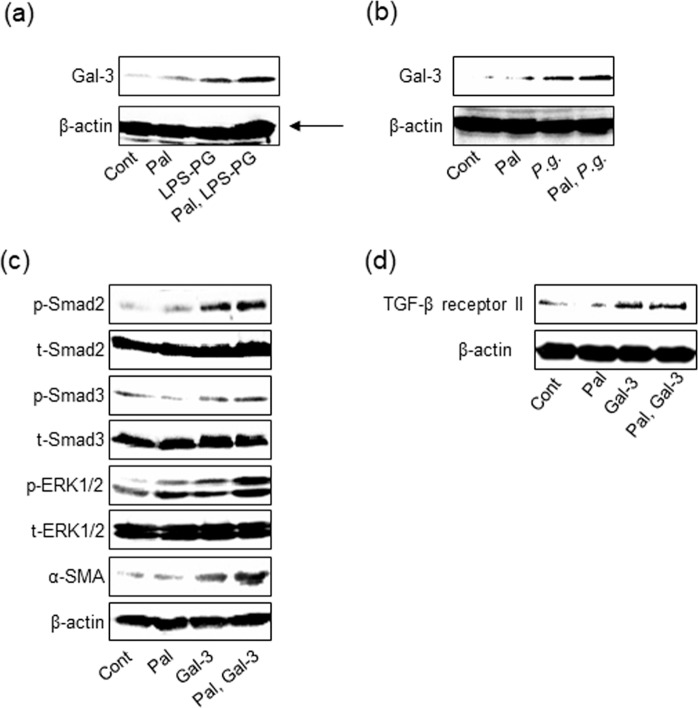
Gal-3 produced from HSCs with *P.g*.-infection or LPS-PG enhances sensitivity to TGF-β1 via upregulating TGF-β receptor II expression. LX-2 cells with/without palmitate treatment were cultured with (**a**) *P.g*. (MOI-100) or (**b**) LPS-PG (1 μg/ml) for 2 days. Gal-3 expression was analyzed by western blotting. (**c**) LX-2 cells with/without palmitate treatment were cultured with recombinant human Gal-3 (1 µg/ml) for 3 days. α-SMA, Smad2, Smad3, and ERK1/2 were detected by western blotting. (**d**) LX-2 cells with/without palmitate treatment were cultured with Gal-3 (1 µg/ml) for 2 days. TGF-β receptor II was detected by western blotting. β-actin was used as internal control. Pal: palmitate, Gal-3: Galectin-3.

To clarify the mechanism in which LPS-PG stimulation induced myofibroblastic differentiation of HSC, we focused on Gal-3, which is one of the most important molecules to stimulate liver fibrosis^16,20^. LX-2 cells markedly induced Gal-3 production by LPS-PG [Fig. 5a]. Interestingly, Gal-3 expression was also upregulated by *P.g*. infection [Fig. 5b]. The direct effect of Gal-3 on LX-2 cells was examined. Gal-3 upregulated α-SMA expression in LX-2 cells through Smad and ERK pathways as well as TGF-β1 [Fig. 5c]. It is known that signaling starts with TGF-β binding to TGF-β receptor II and phosphorylates TGF-β receptor I resulting in phosphorylating downstream including Smad2 and 3^26^. Therefore, TGF-β receptor II expression level was examined. Gal-3 upregulated TGF-β receptor II expression of LX-2 with/without palmitate treatment [Fig. 5d]. Collectively, it is suggested that Gal-3 promoted HSC activation via upregulation of TGF-β receptor II expression resulting in increasing the sensitivity for TGF-β1.

### TGF-β1 and Gal-3 production from hepatocytes by *P.g.-*infection and -LPS stimulation contributed to HSC activation in paracrine manner

Hepatocytes are the most common constitutive cells of the liver. To examine the involvement of hepatocytes in myofibroblastic differentiation of HSCs, productions of TGF-β1 and Gal-3 from hepatocytes were analyzed.

**Figure 6:**
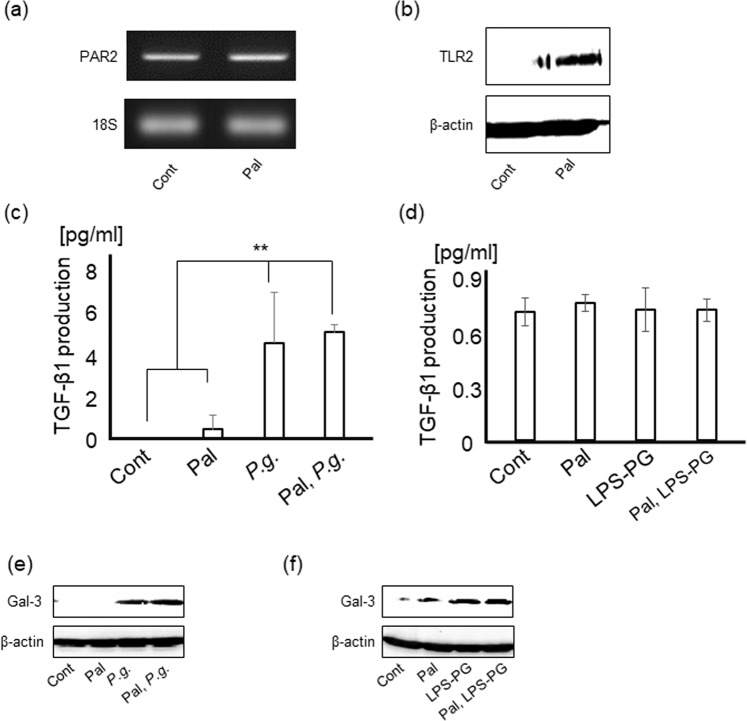
Hepatocytes produce TGF-β1 by *P.g.-*infection and Gal-3 by *P.g*. infection/LPS-PG stimulation. PAR2 (**a**) and TLR2 (**b**) expressions in Hc3716 cells with/without palmitate treatment were detected by RT-PCR and western blotting, respectively. (**c**) Hc3716-hTERT cells with/without palmitate treatment were cultured with *P.g* (MOI-100) for 24 hours. Cont/Pal/*P.g*.: N = 3, Pal, *P.g*.: N = 4. (**d**) Hc3716-hTERT cells with/without palmitate treatment were cultured with LPS-PG (1 μg/ml) stimulation for 24 hours. Cont/Pal/LPS-PG/Pal, LPS-PG: N = 5. The amount of TGF-β1 in each supernatant was measured by ELISA. Hc3176-hTERT cells with/without palmitate treatment were cultured with (**e**) *P.g* (MOI-100) or (**f**) LPS-PG (1 μg/ml) for 2 days. Gal-3 expression was analyzed by western blotting. 18 S was used as internal control for RT-PCR and β-actin was used as internal control for western blotting. Results were shown as mean ± SD. **p < 0.01. Pal: palmitate, *P.g*.: *P.g.-*infection, Gal-3: Galectin-3.

Expression of TLR2 in Hc3716-hTERT cells, immortalized human fetal hepatocytes, was significantly increased by palmitate treatment for 18 hours, however, neither TLR4 [Supplementary Fig. 3b] nor PAR2 levels were increased [Fig. 6a,b]. TGF-β1 from Hc3716-hTERT cells with/without palmitate was also significantly up-regulated by *P.g.-*infection [Fig. 6c], but not by LPS-PG [Fig. 6d]. Further, upregulation of Gal-3 production in Hc3716-hTERT cells was prominently caused by not only *P.g*.-infection [Fig. 6e] but also by LPS-PG stimulation [Fig. 6f]. These data indicate that TGF-β1 and Gal-3 produced from hepatocytes additionally promote myofibroblastic differentiation of HSCs in paracrine manner.

## Discussion

It is well accepted that invasive enterobacteria such as *Escherichia coli* (*E. coli*) and their derived PAMPs play a critical role in pathogenesis of NASH^12,27,28^. Especially, LPS derived from *E. coli* in portal blood reaches the liver and enhances tissue necrosis factor α (TNFα) production from Kupffer cells through TLR4 signaling and leads to pathological progression of NASH. On the other hand, it is suggested that non-invasive bacteria such as *Lactobacillus salivarius*, *acidophilus* and *Pediococcus pentosaceus* do not induce liver fibrosis, or rather prevent liber fibrosis^6,10,29–32^. *Lactobacillus acidophilus* has anti-inflammatory and antifibrotic activities through inhibiting NF-kB and down-regulating expression of TGF-β1, α-SMA and collagen.

As well as *E. Coli, P.g*., a main periodontal pathogen, has various PAMPs including LPS and lipoprotein, which induce proinflammatory cytokines production from various cells. It is reported that *P.g*. enters the blood circulation from periodontal disease sites, disseminates into the whole body, and induces harmful effects on systemic diseases including NASH. Previously, we reported that *P.g.-*odontogenic infection exacerbated inflammation, using HFD-induced NASH mouse model, in which upregulation of serum LPS was evident. Moreover, *P.g*. was detected in the liver^10^. We also reported that NASH cases with *P.g.-*infection in the liver biopsy showed a significantly higher fibrosis score^10^. Interestingly, there was a report describing that the serum AST and ALT levels of 10 NAFLD patients with periodontitis were significantly improved with oral hygiene instructions such as scaling and root planning procedures for 3 months^33^. In this study, *P.g*. infected from dental pulp induced periapical inflammation with neutrophils-infiltration at local site and was detected in the liver as well as our previous study^10^. We demonstrated that *P.g.-*odontogenic infection induced liver fibrosis and increased the number of hCLS, positively correlated with the extent of liver fibrosis^34^. Further, the immunohistochemical analysis of the liver highlighted that pSmad2 (a downstream of TGF-β1-signaling) and Gal-3 were significantly upregulated with *P.g.-*odontogenic infection, indicating these are key molecules for liver fibrosis induced by *P.g*.-odontogenic infection^16,20^.

*P.g*. has many pathogenic factors including fimbria, bacterial DNA, gingipain and LPS^6–10,13,23,24^. It was reported that gingipain, a trypsin-like cysteine protease, activated PAR2 in oral epithelial cells, gingival fibroblasts and immune cells in the periodontal tissue to produce cytokines including IL-6, 8 and MMP-2 resulting in periodontal breakdown^23,24^. Previous study indicated that secreted gingipains from *P.g*. induced TGF-β1 production from gingival fibroblasts^13^. *P.g.-*LPS also plays a key role in inducing inflammation not only at local but also at distant organs through the circulation. However, there is still controversy in receptors for *P.g*.-LPS^6–10^. Some studies suggested that *P.g*.-LPS exhibited an activity mediated by TLR2 though other studies for synthetic lipid A of *P.g*.-LPS have indicated that they are able to activate cells through TLR4 but not TLR2, suggesting that TLR2 activity induced by *P.g*.-LPS might be attributed to a contaminant lipoprotein^8–11,14,35^. Recently, Nativel *et al*. confirmed that *P.g.-*LPS activity was mediated exclusively through TLR4 and it only weakly induced proinflammatory cytokine secretion in mouse models^36^. Therefore, in this study we used LPS-PG including ligands for TLR2 (*P.g*.- lipoprotein) and 4 (*P.g*.-LPS), to focus on pathological significance of TLR2 since TLR2 was significantly upregulated in palmitate treated hepatocytes and in the liver of HFD-feed mouse^10^. Thus, we hypothesized that gingipain and *P.g*.-LPS/lipoprotein might be potential aggravating factors of pathological progression of NASH.

Accumulating data have demonstrated that HSCs are effector cells for liver fibrosis. In response to liver injury, HSC is activated to be a myofibroblastic phenotype, which is highly proliferative and produces type I collagen (HSC activation)^14^. α-SMA and type I collagen, which are induced by TGF-β1/Smad and ERK pathways^14,19,37,38^, are common makers for HSC activation. TGF-β1 is known as one of the most important key mediators of fibrosis in several organs such as the lung, kidney, and liver^14,17,39–41^ resulting from proliferation and differentiation of myofibroblasts through Smad and ERK signaling pathways^22,37^.

In this study, *P.g.-*infection markedly stimulated HSC differentiation including upregulation of α-SMA and type I collagen production through activating Smad and ERK pathways. Moreover, TGF-β1 production was upregulated by *P.g.-*infection. Hence, TGF-β1 induced by *P.g*.-infection is the major molecule for HSC activation. As described the above, accumulating evidence indicated that activation of PAR2 by extracellular serine proteases induced TGF-β1 production from HSCs, moreover, it was demonstrated that TGF-β1 protein expression were decreased in PAR2 knock-out mice resulting in reduced liver fibrosis^25,42^. Therefore, we hypothesized that gingipain-derived from *P.g*. contributed to production of TGF-β1 from HSCs. As we expected, our study demonstrated that gingipain inhibitors completely inhibited TGF-β1 production from HSCs. Furthermore, palmitate is a major FFA in serum of NASH patients and palmitoylation is critical for efficient PAR2 signaling^43^. In this study, we elucidated that PAR2 was markedly upregulated with palmitate treatment and it significantly increased TGF-β1 production from HSCs with *P.g.-*infection. Gingipain inhibitors reduced TGF-β1 production. Moreover, TGF-β receptor I inhibitor suppressed HSC activation caused by *P.g.-*infection. It is suggested that PAR2 activation caused by gingipain results in liver fibrosis through HSC activation. The possibility may remain that infected *P.g*. include quite small amount of LPS, but the amount was supposed to be too small to have effects on cells, which could be ignored^44,45^.

In addition, HSC proliferation with/without palmitate treatment was significantly promoted by *P.g*.-infection. Proliferation rate of palmitate-treated HSC showing increased activation of gingipain/PAR2/TGF-β1 axis was similar to that of non-treated HSC. It is suggested that other molecules than TGF-β1 induced by *P.g*.-infection may also contribute to HSC proliferation. To clarify the significance of gingipain induced TGF-β1 in *P.g.-*odontogenic infection NASH mouse model, *in vivo* experiments using gingipain inhibitors are needed in near future.

Gal-3 is reported to be another key molecule for liver fibrosis through HSC activation^16,20,46^. Serum Gal-3 level was reported to be higher in advanced cases of liver fibrosis^47^. Gal-3 is produced from HSCs by NF-kB activation or by phagocytosis via integrin^16^. Interestingly, *P.g*. is well known to be phagocytosed via integrin α5β1 and α5β3 while *P.g*.-LPS and lipoprotein activate NF-kB pathway through TLR4 and 2, respectively^16,48–50^. Our data showed that Gal-3 was significantly produced from HSCs with *P.g.-*infection and *P.g*.-LPS/lipoprotein stimulation. Moreover, Gal-3 upregulated TGF-β receptor II at protein level in HSCs. Gal-3 cross-links N-glycans on TGF-β receptors including receptor II and delays their removal by endocytosis^21,51^. Gal-3 expression caused by *P.g.-*infection and *P.g.-*LPS/lipoprotein stimulation may contribute to enhancing the sensitivity of HSCs to TGF-β1 by upregulating TGF-β receptor II, resulting in HSC activation. Moreover, in palmitate-treated HSCs, TLR2 expression is upregulated. Therefore, palmitate-treated HSCs tended to produce higher amount of Gal-3 by *P.g.-*LPS/lipoprotein stimulation, resulting in exacerbating liver fibrosis. These results suggest that steatosis induces upregulations of TLR2 expression, which contribute to high sensitivity to gingipain and *P.g*.-LPS/lipoprotein leading to severe inflammation and fibrosis.

Interestingly, hepatocyte, which is major constitutive parenchymal cell of the liver and exists adjacent to HSC, significantly produced TGF-β1 and Gal-3 with *P.g.-*infection and/or *P.g*.-LPS/lipoprotein as well as HSCs. It is indicated that additional production of TGF-β1 and Gal-3 from hepatocytes may induce further activation of HSCs in a paracrine manner. Moreover, palmitate treatment also upregulated TLR2 expression in hepatocyte, which might increase Gal-3 expression in hepatocyte. Thus, it is suggested that the interaction between HSCs and hepatocytes has critical role in HSC activation caused by *P.g.-*infection and -LPS stimulation, especially in fatty liver.

**Figure 7:**
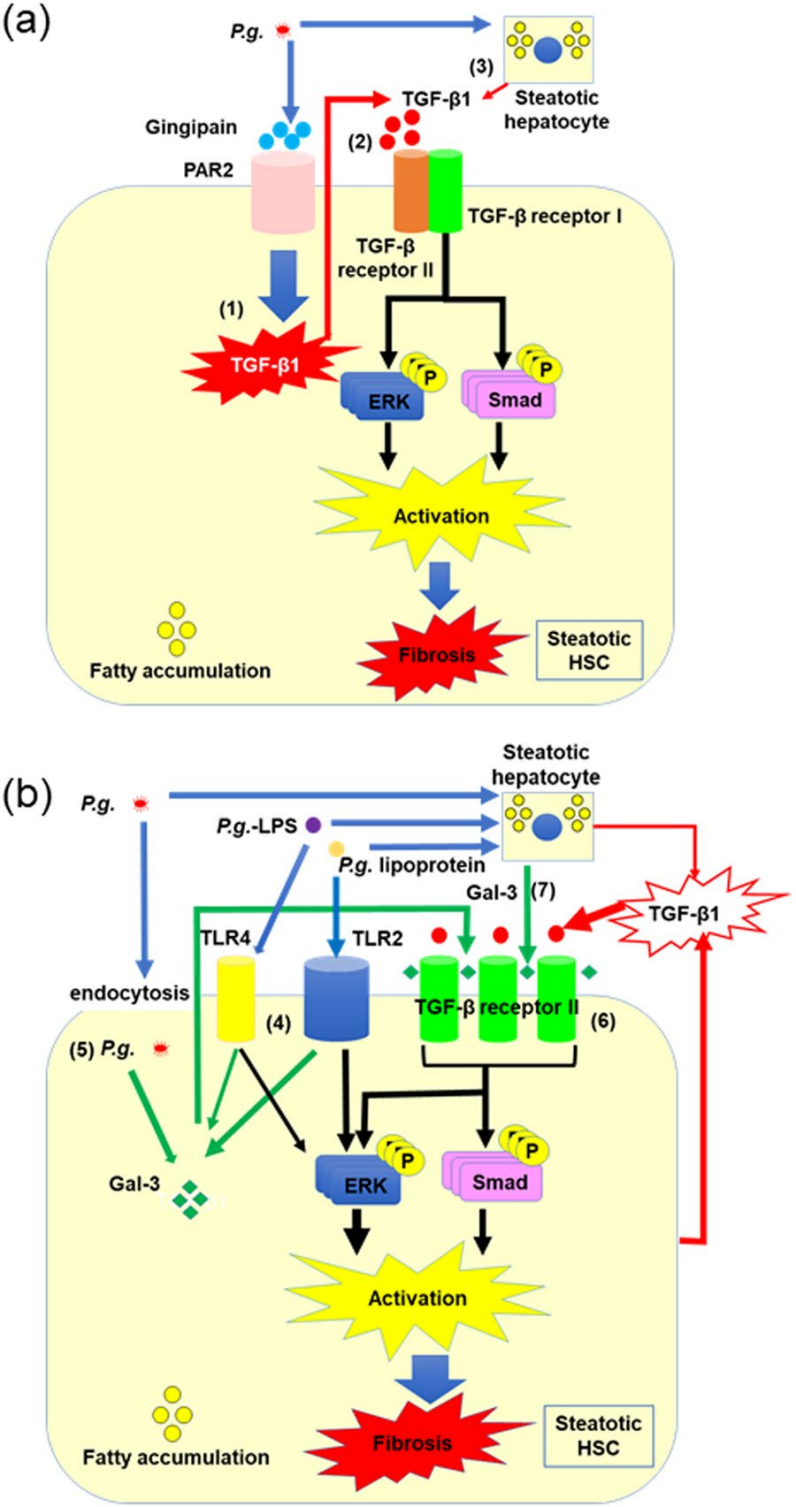
Schematic representation of the characteristic mechanisms of pathological progression of NASH caused by odontogenic infection of *P.g*. (**a**) The mechanisms of HSC activation caused by TGF-β1. (**b**) The mechanisms of HSC activation caused by Gal-3.

With these experimental datasets including *in vivo* and *in vitro*, we suggest the potent mechanisms of HSC activation through TGF-β1 (Fig. 7a) and Gal-3 (Fig. 7b) in steatotic cell. Figure 7(a) PAR2 expression is significantly upregulated with fatty accumulation resulting in excessive TGF-β1 production caused by *P.g.-*infection through gingipain-PAR-2 axis (1). TGF-β1 up-regulates phosphorylation of Smad and ERK via TGF-β receptor I/II complex, leading to marked HSC activation in autocrine manner (2). TGF-β1 produced from steatotic hepatocyte with *P.g-*infection also stimulates HSC activation in paracrine manner (3). Figure 7(b) shows Gal-3-related HSC activation. *P.g.-*lipoprotein induces intense TLR2 signaling via upregulated TLR2-expression caused by fatty accumulation, leading to excessive Gal-3 production together with weak TLR4 signaling by *P.g*.-LPS (4). Gal-3 was also produced with *P.g*. endocytosis by infection (5). Secreted Gal-3 promotes HSC activation through Smad and ERK pathways in autocrine manner. In the mechanism, formation of bridges between TGF-β receptor II and Gal-3, which may keep TGF-β receptor II on the cell surface for longer than usual, eventually resulting in enhanced sensitivity to TGF-β1 (6). Steatotic hepatocyte with *P.g*. infection and/or *P.g*.-LPS/lipoprotein stimulation also produces Gal-3, which accelerates HSC activation in paracrine manner (7).

### Conclusion

The present study indicates that *P.g.-*odontogenic infection exacerbates pathological progression of NASH by stimulating activation of HSCs through TGF-β1 and Gal-3 production. Moreover, TGF-β1 and Gal-3 production from *P.g*. infected and/or *P.g.-*LPS/lipoprotein stimulated hepatocytes contribute to pathological progression of NASH.

## Methods

Please refer to the Supplementary Materials and Methods for more detailed descriptions.

### Animal studies

This study was performed in strict accordance with the recommendations in the Guide for the Care and Use of Laboratory Animals of the Hiroshima University Animal Research Committee and American Veterinary Medical Association (AVMA) Guidelines on Euthanasia. The experimental protocol was approved by the animal care committee of Hiroshima University (A16-58). HFD was fed to 10 mice (HFD-60; Oriental Yeast Co., Ltd., Tokyo, Japan) for 12 weeks to induce fatty liver. Then, the mice were segregated into 2 groups (with or without *P.g.-*odontogenic infection). *P.g*. W83 strain was dentally applied to 5 mice following the same procedure as previously described and 5 mice were named as HP^10^. The remaining 5 mice without *P.g*.-infection were named as HFD. After 9 weeks of *P.g.-*infection, body weights were measured and tissue samples such as periodontal tissue and liver were taken for histological analysis.

### Histological analysis and immunohistochemistry

Paraffin sections of 4.5 (liver tissue) or 6.0 (periodontal tissue) μm thickness were used for histological and immunohistochemical analysis. The fibrosis areas were evaluated with sirius red staining, which visualizes collagen. Immunohistochemical staining (IHC) was performed as previously described^10^. Localization of neutrophils and macrophages was analyzed with Ly-6B.2 alloantigen anti mouse (MCA771GA), (BioRad Laboratories Inc., California, USA) and purified anti-mouse/human Mac-2 (Gal-3), (BioLegend Inc., California, USA), respectively. pSmad2 was detected with Phospho-Smad2 (Ser465/467), (SIGMA-ALDRICH, St. Louis, MO, USA). For *P.g*. detection, anti-*P.g*. polyclonal antibody provided by Professor. Kazuyuki Ishihara (Tokyo Dental University Microbiology Course, Tokyo, Japan) and HistoGreen, Substrate kit for peroxidase (Eurobio Ingen, Les Ulis, France) were used.

### Morphometry

The number of hCLS, which were Mac-2 positive macrophage aggregates, was counted [Supplementary Fig. 2] at randomly selected 5 different fields in each liver section under 200 magnification (gross area; 3.0 × 10^6^ μm^2^). The sirius red positive fibrosis areas were captured as same method as hCLS and measured with image processing software “Image J” (https://imagej.nih.gov/ij/).

### Cell culture

A commonly used human hepatic stellate cell line (LX-2), originally provided by Dr. Tomohiro Ogawa (Kindai University, Hiroshima, Japan), and immortalized human fetal hepatocytes (Hc3716-hTERT), established and provided by Professor. Hidetoshi Tahara (Hiroshima University, Hiroshima, Japan), were used in the present study^52^.

### Cell treatment

Please refer to the Supplementary Materials and Methods for more detailed descriptions. In order to clarify the mechanism of pathological progression of NASH, palmitate, which was FFA to be the major mediators of excessive hepatic lipid accumulation and elevated in circulation of NASH patients, was used in this study, following the previous study^10,53^. In brief, each cell line was cultured in medium containing palmitate (0.2 mM) for 18 hours to induce accumulation of lipids mimicking a fatty liver^10,53^. FFA-free BSA-treated cells were used as control. The cells were incubated in fresh medium without or with *P.g*. infection at multiplicity of infection (MOI) 100, along with *P.g*.-LPS/lipoprotein (1 μg/ml), which is a TLR2 and TLR4 ligand, (LPS-PG; InvivoGen, California, USA), human TGF-β1 (10 ng/ml; R&D Systems, Minnesota, USA), and Gal-3 (1 µg/ml; Peprotech, New Jersey, USA) recombinant protein. For gingipain inhibition, KYT-1 and -36 (Peptide Institute, Inc. Osaka, Japan) were used. KYT-1 and -36 are widely used to inhibit gingipain RgpA, RgpB (arginine-specific cysteine proteinase) and Kgp (lysine-specific proteinase), respectively^54–56^. In this study, KYT-1 and -36 were used each at 3 μM. They were added to the media Dulbecco’s modified Eagle’s medium (DMEM) and mixed with *P.g*. (MOI 100) for 5 minutes before infection. TGF-β receptor I inhibitor (TGF-β RI kinase inhibitor VI, 1 μg/ml), (SB431542, EMD Chemicals, Inc., San Diego, USA) was added to media (DMEM) before 24 hours from *P.g*. infection or TGF-β1 stimulation. Then HSCs were cultured with *P.g*. (MOI 100) or TGF-β1 (1 ng/ml) in the media for 24 hours.

### Bacterial strains and culture conditions

*P.g*.-W83 strain was incubated at 37 °C under anaerobic conditions by using AnaeroPack (Mitsubishi Gas Chemical Co., Tokyo, Japan) for 4 days as previously described^10^. Optical density (OD) was measured at 660 nm using a spectrophotometer to count *P.g*. (Spectronic 200), (Thermo Fisher Scientific, Kanagawa, Japan) suspended in Phosphate Buffered Saline (PBS) for measuring it.

### Proliferation assay

The effect of *P.g*. infection and *P.g*.-LPS to affect cell proliferation was determined using a coulter counter (Beckman Coulter Z1; BECKMAN COULTER Life Sciences, Tokyo, Japan) at day 5 after with/without *P.g*. infection or with/without LPS-PG, following manufacturer’s directions.

### RNA isolation and reverse transcription polymerase chain reaction (RT-PCR)

Reverse transcription polymerase chain reaction (RT-PCR) protocol and the PCR primer sequences used are provided as Supplementary Information.

### Western blotting

Protocols as well as details of the primary and secondary antibodies used in the study are provided in the Supplementary Information.

### Enzyme-linked immunosorbent assay (ELISA)

Cells were infected with *P.g*. at MOI 100 or stimulated with LPS-PG at 1 µg/ml for each experimental time point. Protein levels of TGF-β1 in the supernatant were analyzed by using a human TGF-β1 immunoassay kit (Duo Set, R&D Systems) following manufacturer’s instructions.

### Statistical analysis

Results are reported as mean ± standard deviation (SD). Differences among groups were evaluated with one-way ANOVA followed by Tukey’s post-test using SSRI for windows (Social Survey Research Information Co., Ltd., Tokyo, Japan). The level of significance is described as *p* < 0.01 (**) and *p* < 0.05 (*).

## Supplementary information

Supplementary information

## Data Availability

Any restrictions on the availability of materials or information are disclosed.
