# Supplementary material for: Odontogenic infection by Porphyromonas gingivalis exacerbates fibrosis in NASH via hepatic stellate cell activation
Source: Sci Rep. 2020 Mar 5;10:4134. doi: 10.1038/s41598-020-60904-8 (PMC7058079; doi:10.1038/s41598-020-60904-8)
Supplement: Supplementary file 1 — Supplementary information [file 41598_2020_60904_MOESM1_ESM.pdf]

Odontogenic infection by *Porphyromonas gingivalis* exacerbates fibrosis in NASH via hepatic stellate cell activation

Atsuhiro Nagasaki[1], Shinnichi Sakamoto[1], Chanbora Chea[1], Eri Ishida[2], Hisako Furusho[1], Makiko Fujii[3], and Takashi Takata[1,4]*, Mutsumi Miyauchi[1]*

[1]Department of Oral and Maxillofacial Pathobiology, Graduate School of Biomedical and Health Sciences, Hiroshima University, Hiroshima, Japan

[2]Department of Advanced Prosthodontics, Graduate School of Biomedical and Health Sciences, Hiroshima University, Hiroshima, Japan

[3]Department of Global Dental Medicine & Molecular Oncology, Graduate School of Biomedical and Health Sciences, Hiroshima University, Hiroshima, Japan

[4]Tokuyama University, Tokuyama, Japan

*Corresponding authors: Mutsumi Miyauchi (key corresponding author) and Takashi Takata

Supplementary information (data, materials and methods)

Supplementary data

Supplementary Figure 1. Effects of *P.g.*-odontogenic infection on mouse body weights.

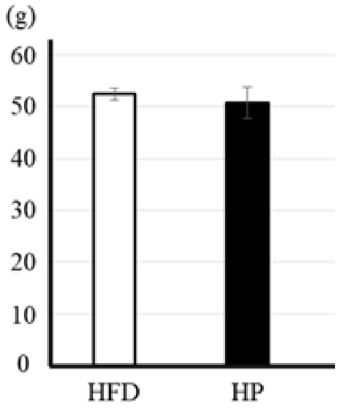

During this study, body weights were not significantly different between each group.

Body weights were recorded until collecting samples.

Supplementary Figure 2. Histopathological feature of hepatic crown-like structures (hCLS)

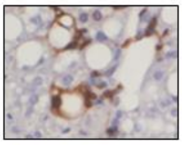

Hepatic crown-like structures (hCLS): A macrophage aggregation surrounds large lipid droplets resulting from hepatocyte death, a common histological feature for NASH, and positively correlates with the extent of liver fibrosis. hCLS were labeled with MAC2.

Supplementary Figure 3. Effect of palmitate treatment on toll like receptor 4 (TLR4) expression in hepatic stellate cell (HSC) (LX-2; human hepatic stellate cell line) and hepatocyte (Hc3716; human hepatocyte cell line).

(a)

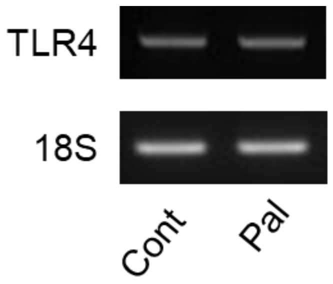

LX-2 cells were pretreated with/without 0.2 mM of palmitate for 18 h. There was no significant difference between control vs. palmitate treatment.

(b)

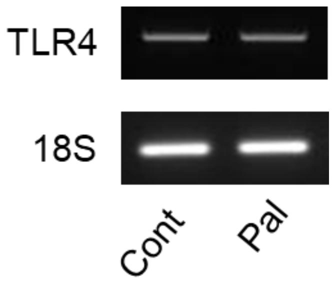

Hc3716 cells were pretreated with/without 0.2 mM of palmitate for 18 h. There was no significant difference between control vs. palmitate treatment.

Pal: palmitate

Supplementary Figure 4. Effects of TGF-β1 on myofibroblastic differentiation of HSC.

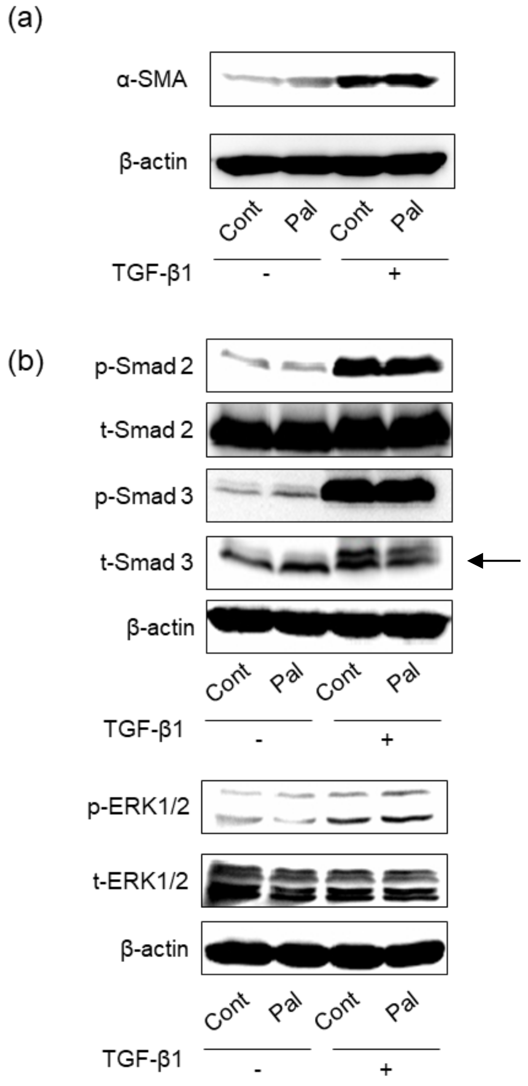

α-SMA is prominently upregulated and Smad2, Smad3, and ERK1/2 pathways are activated with TGF-β1 stimulation.

LX-2 cells were pretreated with/without 0.2 mM of palmitate for 18 h. (a) LX-2 cells with/without palmitate treatment were cultured with TGF-β1 (10 ng/ml) for 6 days. α-

SMA was detected by western blotting. (b) LX-2 cells with/without palmitate treatment were cultured with TGF-β1 (10 ng/ml) for Smad2, Smad3, and ERK1/2. Smad2, Smad3, and ERK1/2 were detected by western blotting. β-actin was used as internal control. Pal: palmitate

Data availability

Any restrictions on the availability of materials or information are disclosed.

Animal study

For *P.g.*-odontogenic infection, the roofs of the pulp chambers of the right and left first molars of the maxilla were removed with #1/2 round bar (Morita Co., Osaka, Japan) under intraperitoneal anesthesia with (Pentbarbital sodium: 1.94 mg/50 g, Kyoritsu Seiyaku Co., Tokyo, Japan) and atropine sulfate (15 μg/50 g, Fuso Pharmaceutical Industries, Ltd., Osaka, Japan). After removing the coronal pulp, a small cotton swab with 1 μl of PBS containing 10^7 colony-forming units (CFU) of *P.g.* W83 strain was inserted into the pulp chamber and sealed with Caviton (GC Co., Tokyo, Japan).

Histological analysis and Immunohistochemistry

PLP solution consists of 0.1 M phosphate buffer (Sigma-Aldrich Japan Co., Tokyo, Japan), 0.2 M Lysine HCl (Sigma-Aldrich Japan Co.), and 8 % paraformaldehyde (Merck KGaA, Darmstadt, Germany). Tissue samples were fixed in PLP solution for 48 h, processed and embedded in paraffin. The periodontal tissues were immersed in decalcifying solution consisting of ethylenediaminetetraacetic acid disodium salt dihydrate and ethylenediaminetetraacetic acid tetrasodium salt, tetrahydrate (Sigma-Aldrich Japan Co.) for 4-weeks before paraffin-embedding. The liver and periodontal tissue samples were cut at 4.5 μm and 6.0 μm thickness, respectively. Sirius red staining was performed for measurement of fibrosis areas. In brief, the slides were stained with sirius red solution (Wako Pure Chemical Industries, Ltd., Osaka, Japan) for

1 h at room temperature after deparaffinization. Then, they were washed with glacial acetic acid (Sigma-Aldrich Japan Co.) twice. Then they were covered with slide glasses after dehydration.

Immunohistochemical staining (IHC) was performed as previously described (Furusho H *et al.* 2013). The tissue sections were deparaffinized and immersed in methanol with 0.3 % hydrogen peroxide. The liver sections for IHC were incubated with primary antibodies Mac-2 (Galectin-3), (BioLegend Inc., California, USA) and Ly-6B.2 (Bio-Rad, Virginia, USA) at 4 °C overnight. The staining was visualized by using a DAB Peroxidase (HRP) Substrate Kit (DAKO Japan, Tokyo, JAPAN) to detect the antigen-antibody reactions.

Cell culture

A human hepatic stellate cell line (LX-2), (kindly provided by Dr. Tomohiro Ogawa, Kinki University, Japan) was cultured in Dulbecco's modified Eagle's medium (DMEM) media (Nissui Pharmaceutical Co., Ltd. Tokyo, Japan) supplemented with 10 % heat-inactivated FBS (Invitrogen, Life Technologies Japan Ltd, Tokyo, Japan) and 100 U/ml penicillin–streptomycin (Gibco, Tokyo, Japan). Immortalized human fetal hepatocytes (Hc3716-hTERT), (kindly provided by Professor Hidetoshi Tahara, Chair of Department of Cellular and Molecular Biology, Institute of Biomedical & Health Sciences, Hiroshima University,

Japan) were maintained in Hepatocyte Basal Medium (HBM; Lonza, Walkersville, USA) with 15 % heat-inactivated FBS (Invitrogen, Life Technologies Japan Ltd.) as described previously in CELL STAR dishes (Greiner Bio-One GmbH, Frickenhausen, Germany), (Furusho H *et al.* 2013). For experiments, type I collagen-coated cell culture dishes were used for Hc3716 (MS-0060K, MS-0096K), (Sumitomo Bakelite Company Limited, Tokyo, Japan). Cells were maintained at 37 °C in 5 % CO_2. For experiments, cells were seeded at a density of 4 $\times 10^3$ cells in a 6-well culture dish for proliferation assay, 5×10^3 cells in a 96-well culture dish for enzyme-linked immunosorbent assay (ELISA) and 30×10^4 cells in a 60-mm culture dish for western blotting analysis and reverse transcription polymerase chain reaction.

Palmitate treatment

Palmitate (100 mM, Sigma-Aldrich Co., St. Louis, USA) and 10 % bovine serum albumin (BSA; Sigma-Aldrich Co.) stock solution were prepared according to the previously described method (Wobser H *et al.* 2009). Palmitate stock solution (100 mM) was prepared in 0.1 mM NaOH by heating at 70 °C for 15 min and stored at -20 °C until use. BSA solution (10 %) was prepared in ddH_2O and maintained at 55 °C in a water bath for 15 min and stored at -20 °C. Palmitate 10 mM/ 10 % BSA solution was obtained by mixing the appropriate amount of palmitate stock solution to 10 %

BSA at 55 °C for 15 min. The above solutions were then cooled to 25 °C and sterilized using membrane filter. A filter (0.25 μm, Nippon Genetics Co., Ltd., Tokyo, Japan) was used for BSA. A filter (0.45 μm, Merck Millipore Ltd., County Cork, Ireland) was used for palmitate sterilization.

LX-2 cells and Hc3716-hTERT cells were cultured in each medium containing 0.2 mM palmitate for 18 h to induce accumulation of lipids mimicking a fatty liver. FFA-free-BSA-treated cells were used as control.

RNA isolation and reverse transcription polymerase chain reaction (RT-PCR)

Total RNA was extracted from the cells using TRIzol reagent (Invitrogen, Tokyo, Japan) and quantified by using standard spectrophotometric methods. Briefly, complimentary DNA (cDNA) was synthetized from 1 μg of total RNA with a Reva Tra Ace kit (TOYOBO Biochemicals, Tokyo, Japan). For normalization, 18 S rRNA was used as an internal control. PCR primer sequences are listed below. Human 18S: Forward; 5'-ACACGGACAGGATTGACAGA-3', Reverse; 5'-CAAATCGCTCCACCAACTAA-3', Human Protease-activated receptor 2 (PAR2): Forward; 5'-AGAAGCCTTATTGGTAAGGTT-3', Reverse 5'-AACATCATGACAGGTCGTGAT-3', Human Toll like receptor 4 (TLR4): Forward; 5'-CTGCAATGGATCAAGGACCA-3', Reverse 5'-TCCCACTCCAGGTAAGTGTT-3'.

Western blotting

Cells were washed with ice-cold PBS and lysed in buffer containing 0.1 % Triton X-100 (Roche, Castle Hill, Australia), 10 μg/ml L-1 chlor-3-(4-tosylamido)-4 phenyl-2 butanon (TPCK), 1 mM DTT, 0.1 mM Na_3VO_4, 10 μg/ml L-1 chlor-3-(4-tosylamido)-7-amino-heptanon-hydrochloride (TLCK), 0.1 mM leupeptin and 50 μg/ml phenylmethylsulfonyl fluoride (PMSF) for 30 min. The lysates were centrifuged at 13,200 rpm for 20 min at 4 °C. Supernatants were collected as protein lysate and determined by Bradford protein assay (Bio-Rad, Virginia, USA). The samples were then prepared in Laemmli buffer at 100 °C for 3 min and separated by 10 % polyacrylamide gel (PAGE) electrophoresis and transferred to nitrocellulose membrane (Schleicher & Schuell, Dasse, Germany). The membranes were then blocked using 5 % milk for 1 h at 25-28 °C room temperature prior to application of primary antibodies and incubated at 4 °C overnight.

The following antibodies were used as primary antibodies; anti-human smooth muscle actin clone 1A4, (Dako, Glostrup, Denmark), anti-collagen I (ab34710), anti-toll like receptor (TLR) 2, (ab68159), (abcam Japan, Tokyo, Japan), Mac-2 (Galectin-3), (BioLegend Inc., California, USA), anti-phospho-ERK1/2 (#4376), anti-ERK1/2 (#4695), anti-phospho-Smad2 (#3108), anti-Smad2 (#5339), anti-phospho-Smad3 (#9520), and

anti-Smad3 (#9523), (Cell Signaling Technology Japan, K.K., Tokyo, Japan). Mouse monoclonal anti-β-actin, (Sigma-Aldrich Co, Missouri, USA) was used as an internal control. Anti-mouse antibody (#7076), anti-rabbit antibody (#7074), (Cell Signaling Technology Japan, K.K.) and anti-rat antibody (A18865), (Life Technologies Ltd., Massachusetts, USA) were used as secondary antibodies.

The ECL western blotting detection system (Western Lightning ECL Pro), (PerkinElmer, INC., Massachusetts, USA) was used for visualization.

Data availability

Any restrictions on the availability of materials or information are disclosed.
